# *Bacillus pumilus* KatX2 confers enhanced hydrogen peroxide resistance to a *Bacillus subtilis* P*katA::katX2* mutant strain

**DOI:** 10.1186/s12934-017-0684-y

**Published:** 2017-04-26

**Authors:** Stefan Handtke, Dirk Albrecht, Daniela Zühlke, Andreas Otto, Dörte Becher, Thomas Schweder, Kathrin Riedel, Michael Hecker, Birgit Voigt

**Affiliations:** 1 Institute for Microbiology, University of Greifswald, 17489 Greifswald, Germany; 2 Institute of Pharmacy, University of Greifswald, 17489 Greifswald, Germany; 3 Institute of Marine Biotechnology, 17489 Greifswald, Germany; 4 Present Address: Research Institute for Leather and Plastic Sheeting, Meißner-Ring 1-5, 09599 Freiberg, Germany

**Keywords:** *Bacillus pumilus*, Hydrogen peroxide, Oxidative stress, Catalase

## Abstract

**Background:**

*Bacillus pumilus* cells exhibit a significantly higher resistance to hydrogen peroxide compared to closely related Bacilli like *Bacillus subtilis*.

**Results:**

In this study we analyzed features of the catalase KatX2 of *B. pumilus* as one of the most important parts of the cellular response to hydrogen peroxide. KatX2, the vegetative catalase expressed in *B. pumilus*, was compared to the vegetative catalase KatA of *B. subtilis*. Data of our study demonstrate that *B. pumilus* can degrade toxic concentrations of hydrogen peroxide faster than *B. subtilis*. By replacing *B. subtilis* *katA* gene by *katX2* we could significantly enhance its resistance to H_2_O_2_ and its potential to eliminate this toxic compound. Mutant cells showed a 1.5- to 2-fold higher survival to toxic concentrations of hydrogen peroxide compared to wild type cells. Furthermore, we found reversible but also irreversible oxidations of the KatX2 protein which, in contrast to KatA, contains several cysteine residues.

**Conclusions:**

Our study indicates that the catalase KatX2 plays a major role in the increased resistance of *B. pumilus* to oxidative stress caused by hydrogen peroxide. Resistance to hydrogen peroxide of other Bacilli can be enhanced by exchanging the native catalase in the cells with *katX2*.

**Electronic supplementary material:**

The online version of this article (doi:10.1186/s12934-017-0684-y) contains supplementary material, which is available to authorized users.

## Background

In their natural habitat (soil) as well as in industrial fermentation processes cells are frequently exposed to different stress conditions like heat, osmotic or oxidative stress [1–7]. Oxidative stress can be caused by a variety of reactive oxygen species (ROS) like superoxide (O_2_^·−^), hydrogen peroxide (H_2_O_2_) and hydroxyl radical (OH·). Such conditions may interfere with the fermentation processes by hampering the cell growth, killing the cells or by impairing the quality of the fermentation product.

*Bacillus pumilus* strains show a significantly increased resistance to oxidative stress caused by hydrogen peroxide compared to its relatives belonging to the *Bacillus subtilis* species complex [8–10]. A mechanistic understanding of this phenomenon could help to improve the oxidative stress resistance of other production strains which are already in use.

Catalases are key enzymes for the degradation of hydrogen peroxide. In many well-known *Bacillus* species KatA is the primary catalase expressed in vegetative cells [11, 12]. The genome of *B. pumilus* does not encode any gene homolog of KatA [13]. Instead, it encodes two genes annotated as catalases KatX1 and KatX2. KatX1 shows a homology of 83% to *B. subtilis* KatX, the spore catalase of this organism [14]. KatX2 shows only about 50% homology to both the KatX and the KatA amino acid sequence of *B. subtilis*. Like KatA, KatX2 is an iron dependent catalase.

In this study and also in previous studies using *B. pumilus* Jo2 we never observed an expression of *katX1*, neither on the proteome nor on the transcriptome level. In contrast, KatX2 showed high induction rates on both levels following H_2_O_2_ treatment in *B. pumilus* Jo2 [8]. This indicates that KatX2 may take on the role as the major vegetative catalase in *B. pumilus*.

In this study we compared the abundance and the activity of the catalase from *B. pumilus* with those of related organisms like *B. subtilis*. We replaced *B. subtilis* *katA* gene by the *B. pumilus katX2* gene and studied the resistance of this mutant to hydrogen peroxide. Furthermore, since KatX2 contains several cysteine residues, we analyzed the oxidation of this protein under control conditions and during hydrogen peroxide provoked oxidative stress.

## Methods

### Strains, media and growth

*Bacillus pumilus* SAFR-032 (Gioia [13]) and *B. subtilis* 168 [15] were used in this study. Cells were grown aerobically at 37 °C and 180 rpm in a chemically defined medium containing 15 mM (NH_4_)_2_SO_4_, 8 mM MgSO_4_ × 7H_2_O, 27 mM KCl, 7 mM Na-citrate × 2H_2_O, 50 mM Tris–HCl (pH 7.5) supplemented with 1.8 mM KH_2_PO_4_, 2 mM CaCl_2_, 1 µM FeSO_4_ × 7H_2_O, 10 µM MnSO_4_ × 4H_2_O, 4.5 mM glutamate, 62.4 µM tryptophan, 0.2% w/v glucose and 0.04 µM biotin. All growth experiments were done in triplicates.

### Construction of mutant strains

A linear DNA fragment carrying a 600 kb upstream-fragment with the promoter region including the ribosome binding site of *B. subtilis* *katA*, the *B. pumilus katX2* gene from the ATG start-codon to the stop-codon, the spectinomycin resistance gene from pUS19 [16] and a 600 kb downstream fragment beginning right behind the *B. subtilis* *katA* stop codon was created with a two-step fusion PCR (Additional file 1: Figure S1) [17]. Firstly, single DNA fragments of the upstream- and downstream-region of *B. subtilis* *katA*, the *B. pumilus katX2* gene and the spectinomycin resistance gene were created using chromosomal DNA of *B. subtilis* 168, *B. pumilus* SAFR-032 or the pUS19 plasmid and primers extended by several nucleotides homologous to the connecting upstream/downstream-fragment (Table 1). PCR-fragments were separated from template and primer DNA by electrophoresis in a 0.8% agarose gel, cut out in the absence of UV irradiation and purified using the Qiaquick Gel Extraction Kit (Qiagen, Germany). The upstream fragment and *B. pumilus katX2* gene as well as the spectinomycin resistance gene and the downstream fragment were fused through their complementary ends and fusion products were purified as described above. In a second fusion step the upstream-*katX2* fragment and the spectinomycin resistance-downstream fragment were fused to create the complete linear DNA fragment. It was purified as described above. The purified linear fragment was used for transformation of naturally competent *B. subtilis* cells [18, 19].

**Table 1:** Primers used for the amplification and fusion of the *katX2* gene with the spectinomycin resistance gene and the flanking homologous sequences

	Name	Sequence (5′–3′)
1	katA up600 fw	GCGGTGTTCCTGAAAAATAA
2	katA up600 rev	TGAATTTGTCATGTTATCACCTCTTGGAATTTATAAGAAC
3	katX2 fw	AGAGGTGATAACATGACAAATTCAAATCATAAAAATTTG
4	katX2 rev	TTGTTAATTAAATCAATTATTTCATGTTTCCTTGAAGGTAT
5	spec FW	GAAACATGAAATAATTGATTTAATTAACAACTATGGATATAAAATAG
6	spec rev	TGCATTTCTCCATTATTATAATTTTTTTAATCTGTTATTTAAATAGTTTATAGTT
7	katA down600 fw	TAAAAAAATTATAATAATGGAGAAATGCAAAAACC
8	katA down600 rev	TTAAAAGGGAAAAGTTCTCATAGC

Naturally competent *B. subtilis* cells were created by cultivation of *B. subtilis* in SPI (chemically defined medium containing 15 mM (NH_4_)_2_SO_4_, 80 mM K_2_HPO_4_, 40 mM KH_2_PO_4_, 3,5 mM trisodium citrate, 0.8 mM MgSO_4_, 0.02% w/v casamino acids, 0.4% w/v yeast extract, 0.5% w/v glucose) at 37 °C until the end of logarithmic growth followed by 1.5 h cultivation of 10 ml of the culture in 90 ml fresh SPI at 37°.

Transformation was carried out by incubating of 1 µg purified linear DNA with 1 ml competent cells for 1 h in a 100 ml shake flask at 37 °C and 70–100 rpm. 5 ml of LB was added and cells were incubated for 1.5 h at 37 °C and 200 rpm.

Mutants were selected on LB agar plates containing 200 µg/ml spectinomycin. For the verification of the mutation, chromosomal DNA was amplified and sequenced by Eurofins (http://www.eurofinsgenomics.eu/de/home.aspx).

### Sample preparation

Cells were harvested or stressed at an OD_500_ of 0.6 with various concentrations of H_2_O_2_. Samples used for 2D-PAGE analyses, fluorescence thiol modification assays and the quantification of catalase protein accumulation were stressed with 50 µM (*B. subtilis*, *B. subtilis* P*katA::katX2*,) and 2 mM (*B. pumilus*) H_2_O_2_, respectively. Samples used for the catalase activity assay were harvested at an OD_500_ of 0.6 or stressed using one-tenth of these concentrations for 10 min and then harvested.

Cells were harvested by centrifugation (20,000×*g*, 4 °C, 10 min) followed by two washing steps with 100 mM Tris–HCl buffer, pH 7.5. Cell disruption was done by sonication after resuspension in TE buffer (10 mM Tris, pH 7.5, 10 mM EDTA) containing 1.4 mM PMSF. For absolute protein quantification an in-solution digestion of proteins with TE buffer without PMSF was used. Protein concentration was determined with RotiNanoquant (Roth).

### Survival assay

The survival of *B. subtilis* 168 and the P*katA*::*katX*2 mutant was analyzed by adding 500 µM H_2_O_2_ to exponentially growing cells at an OD_500_ of 0.6. Cells were diluted using 0.9% NaCl to appropriate concentrations before addition of hydrogen peroxide, 3 and 15 min after hydrogen peroxide addition and plated on LB agar plates (Invitrogen). Colony forming units were counted after overnight incubation at 37 °C.

### 2D-PAGE, gel imaging, relative quantification and protein identification

200 µg protein were adjusted to 306 µl with 2 M thiourea/8 M urea, mixed with 34 µl CHAPS solution (20 mM DTT, 1%  w/v CHAPS, 0.5%  v/v Pharmalyte, pH 4–7 or 3–10) and loaded onto commercially available IPG strips (SERVA Electrophoresis) in the pH-range of 4–7. IEF was performed according to Büttner et al. [20]. Equilibration of the strips containing the focused proteins was performed in solutions containing DTT and iodoacetamide, respectively, as described by Görg et al. [21]. Gels of 12.5% acrylamide and 2.6% bisacrylamide were used for separation in the second dimension. Gels were stained with Flamingo Fluorescent Gel Stain (Bio-Rad Laboratories) according to the manufacturer`s instructions. 2D-PAGE was done with three independent biological replicates.

Analysis of the gel images and spot quantification was performed as described by Wolf et al. using the Delta2D software version 4.4 (Decodon) [22]. Protein spots were excised from the gels (Ettan Spot Picker, GE Healthcare), digested and spotted onto MALDI targets (Ettan Spot Handling Workstation, GE Healthcare). MS-analysis of the targets was performed by MALDI-TOF-MS/MS using the Proteome Analyzer 4800 (Applied Biosystems) and peak lists were searched with MASCOT search engine version 2.1.0.4 (Matrix Science) and search parameters as described by Wolf et al. [22].

### Label-free quantification (LC-IMS^E^)

In-solution digestion of protein extracts with trypsin was done according to the method described previously [23]. Desalting of peptides prior to mass spectrometry analysis using stage tips was achieved using a standard protocol [24]. For absolute quantification the peptide mix was spiked with a tryptic digest of yeast alcohol dehydrogenase (Waters) at a final concentration of 50 fmol/µl.

The nanoACQUITY™ UPLC™ system (Waters) was used to separate the peptide mixture and to introduce the samples into the mass spectrometer. The peptide mixture was directly loaded on an analytical column (nanoACQUITY™ UPLC™ column, BEH300 C18, 1.7 mm, 75 mm_200 mm, Waters). Separation of peptides for IMS^E^ (MS^E^ with ion mobility separation) was done with a 90 min gradient from 5% buffer B to 40% buffer B. All MS^E^ analyses were performed as previously described [23]. The only modification was, that the collision energy was alternated between 4 eV in the precursor ion trace and a ramp 25–45 eV for fragment ion trace. Wave velocity was ramped from 1000 to 400 m/s, wave height was set to 40 V.

LC-IMS^E^ data were processed using PLGS v3.0.1. Processing parameters were set as follows: Chromatographic peak width and MS TOF resolution were set to automatic, lock mass charge 2 set to 785.8426 Da/e with a lock mass window of 0.25 Da, low energy threshold 200.0 counts, elevated energy threshold 20.0 counts, intensity threshold 750 counts. The data were searched against a randomized *B. subtilis* 168 database (NCBI, version August 2014) with added amino acid sequence of *B. pumilus* SAFR-032 KatX2 protein, laboratory contaminants and yeast ADH1 sequence (8438 entries). For positive protein identification the following criteria had to be met: 1 fragment ion matched per peptide, 5 fragment ions matched per protein, 1 peptide matched per protein; 2 missed cleavages allowed, primary digest reagent: trypsin, fixed modification: carbamidomethylation C (+57.0215), variable modifications: deamidation N, Q (+0.9840), oxidation M (+15.9949), pyrrolidonecarboxylacid N-TERM (−27.9949). The protein false discovery rate (FDR) was set to 5%. For the final analysis only 2 peptide identifications were considered. A protein had to be identified in at least two out of 3 technical replicates per time point; this took the FDR on protein level to less than 3%. Three biological replicates for each time point were analyzed.

Data generated by the IMS^E^ mode were corrected for detector saturation effects by implementing a correction factor based on the ion accounting output files that were created for each sample by the PLGS software. The correction factor (cf) was calculated using the following equation.

$$\frac{{\varSigma I{\text{peptide}}}}{{\varSigma I{\text{product}}}} *\frac{1}{m} = cf$$

where Σ*I*peptide and Σ*I*product are the matched peptide/product intensity sums, *m* is the median of the ratios $\frac{{\varSigma I{\text{peptide}}}}{{\varSigma I{\text{product}}}}$ calculated for every protein quantified in a sample.

### Catalase activity assay

Cells were grown to an OD_500nm_ of 0.6 and cytosolic cell extracts were prepared as described above. Right before starting the assay, a working solution of 25 µg/ml lactoperoxidase and 0.5 M dicarboxidine dihydrochloride (both Sigma-Aldrich) was prepared. To determine the degradation of H_2_O_2_ by cytosolic protein extracts, 5 µg of protein extract were filled up with catalase assay buffer (Catalase Assay Kit, BioVision) to 200 µl. H_2_O_2_ was added to a final concentration of 2 mM and the extracts were incubated at 30 °C. To measure the H_2_O_2_ concentration remaining in the culture at certain time points, 25 µl of the cell extract was mixed with 500 µl working solution and absorbance at 450 nm was measured as described [25]. The experiments were done in triplicates.

### Fluorescence thiol modification assay and analysis of protein modifications

Proteins with reversibly oxidized cysteines were visualized using a protocol described by Hochgräfe et al. [26]. Protein extracts were purified and pre-stained with BODIPY (Thermo Fisher Scientific) as described and loaded onto IPG-strips in the pH-range 4–7 (SERVA Electrophoresis). 2D-PAGE was performed as described above in the dark. Following fluorescence scanning of reversibly oxidized proteins (BODIPY staining), the gels were stained with Flamingo Fluorescent Gel Stain (Bio-Rad Laboratories). Spot quantification and MS-analyses were performed as described above. Oxidation was determined by calculating and comparing the volume of Flamingo stained spots (protein amount) to the volume of BODIPY stained spots (expressed as % volume representing the portion of the spot volume of an individual spot of the entire spot volume detected on the gel). Three biological replicates were performed.

For the analysis of possible modifications, protein spots were excised from the gels as described above, destained (0.2 M NH_4_HCO_3_, 30% acetonitrile) and double digested with trypsin and chymotrypsin (both Promega). Peptide extraction was performed by covering the gel pieces with ultra-pure water (prepared with a Sartorius Stedim unit) and 15 min incubation in an ultrasonic water bath. Peptides were detected by LC–MS/MS using an Orbitrap Elite (Thermo Fisher Scientific). Database searches were conducted with the SEQUEST software v28 (rev.12, Thermo Fisher Scientific) against *B. subtilis* 168 and *B. pumilus* SAFR-032 database. Data were analyzed using Scaffold proteome viewer version 4.0.5.

## Results and discussion

### Growth and survival of the *B. subtilis* P*katA::katX2* mutant

We exchanged *B. subtilis* 168 katA by the *katX2* gene from *B. pumilus*. Only the coding region starting with the ATG start codon of the *B. pumilus* KatX2 gene was fused to the *katA*-promoter region of *B. subtilis*.

**Fig. 1:**
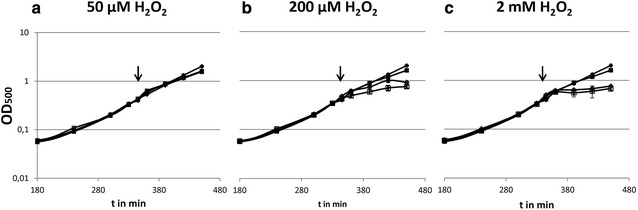
Growth of the *B. subtilis katX2* mutant (*diamonds*) compared to the *B. subtilis* 168 strain (*squares*). Control conditions are shown with *filled*, stressed cultures with *empty symbols*. Time point of addition of H_2_O_2_ is shown by *arrows*. The H_2_O_2_ concentrations used were 50 µM (**a**), 200 µM (**b**) and 2 mM (**c**)

There was no difference in the growth behavior of the *B. subtilis* P*katA::katX2* mutant compared to the wild type strain under control conditions. After the treatment of the wild type strain and the *B. subtilis* P*katA::katX2* mutant with 50 µM H_2_O_2_ also only a small impact on the growth of both strains could be detected (Fig. 1a). In contrast, the mutant strain showed a significantly lower impact on the growth rate than the wild type when the cells were treated with 200 µM H_2_O_2_ (Fig. 1b). Mutant cells continued growth up to an optical density of about 1 whereas the wild type reached a final OD of about 0.75. Increasing the hydrogen peroxide concentration up to 2 mM, a concentration which *B. pumilus* can withstand [8], resulted in a nearly complete cessation of growth in both *B. subtilis* strains (Fig. 1c).

**Fig. 2:**
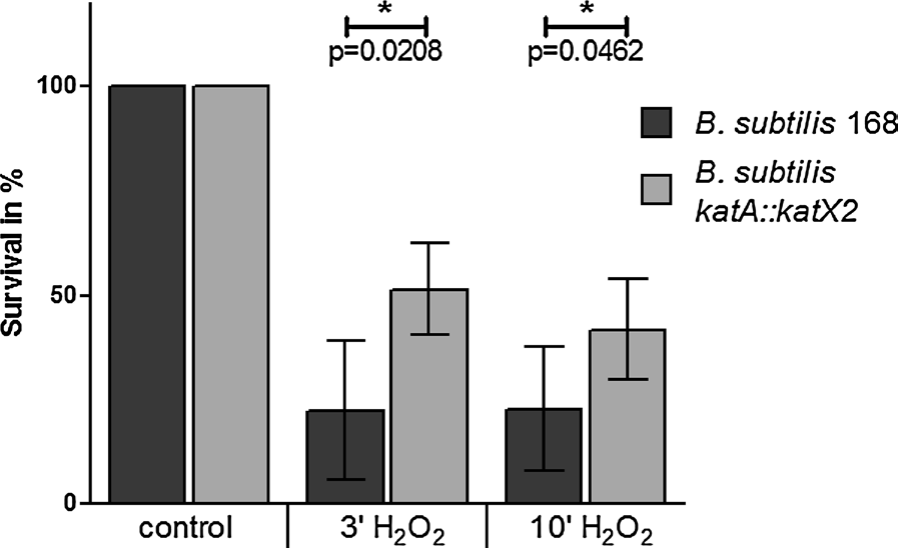
Survival of *B. subtilis* 168 and the *B. subtilis katX2* mutant after 3 and 10 min exposure to 500 µM hydrogen peroxide. The control was set to 100%

The survival of the *B. subtilis* 168 strain and the P*katA::katX2* mutant was analyzed using 500 µM H_2_O_2_. 23% of the wild type cells survived 3 min after peroxide treatment (Fig. 2). At the same time point about 50% of the P*katA::katX2* mutant cells were still alive. 15 min after addition of H_2_O_2_ 32% of the *katX2*-expressing cells were alive whereas only 18% of the wild type cells survived at this time point. These data indicate a 1.5- to 2-fold higher survival of the *katX2*-expressing cells under this oxidative stress conditions.

### Quantification of the catalase protein spots

Two different proteomic approaches were employed to measure cytosolic amounts of catalases KatA and KatX2 in *B. subtilis* and *B. pumilus* in response to hydrogen peroxide stress. First, we analyzed relative changes in the accumulation of KatA and KatX2 catalases following H_2_O_2_ treatment in *B. subtilis* 168, *B. subtilis* P*katA::katX2* and *B. pumilus* using 2D-PAGE.

Under control conditions, the relative spot volumes of the vegetative catalases were 0.37% (KatX2) and 0.39% (KatA) for *B. subtilis* and 0.59% (KatX2) for *B. pumilus* (Table 2). That exponentially growing *B. pumilus* cells contained more catalase than *B. subtilis* cells has been shown before [8]. The KatX2 spot volume only increased about 1.4-fold in *B. pumilus* cells 20 min after H_2_O_2_ treatment. In *B. subtilis* cells we detected a threefold increase from 0.39 to 1.23% in the amount of KatA after H_2_O_2_ treatment indicating that a higher amount of enzyme was present compared to *B. pumilus* cells. A similar about threefold increase after addition of H_2_O_2_ was also observed for the KatX2 spot in the *B. subtilis* P*katA::katX2* mutant.

**Table 2:** Relative spot volume of the protein spots of vegetative catalase KatA or KatX2 on 2D gels

Strain	Control				
	1	2	3	Mean	SD
*B. subtilis* 168					
KatA 1	0.31	0.33	0.32	0.32	0.01
KatA 2	0.04	0.05	0.04	0.05	0.01
KatA 3	0.02	0.03	0.02	0.02	0.00
Sum	0.38	0.41	0.38	0.39	0.01
*B. pumilus* SAFR-032					
KatX2 1	0.10	0.11	0.12	0.11	0.01
KatX2 2	0.09	0.09	0.12	0.10	0.02
KatX2 3	0.09	0.09	0.10	0.10	0.00
KatX2 4	0.26	0.30	0.31	0.29	0.02
Sum	0.54	0.59	0.65	0.59	0.05
*B. subtilis* P*katA::katX2*					
KatX2 1	0.02	0.03	0.03	0.03	0.00
KatX2 2	0.03	0.04	0.03	0.03	0.00
KatX2 3	0.09	0.10	0.09	0.09	0.00
KatX2 4	0.17	0.24	0.25	0.22	0.04
Sum	0.31	0.41	0.40	0.37	0.05

Strain	15′ H_2_O_2_				
	1	2	3	Mean	SD
*B. subtilis* 168					
KatA 1	1.03	0.98	1.10	1.04	0.05
KatA 2	0.13	0.11	0.15	0.13	0.02
KatA 3	0.05	0.05	0.09	0.06	0.02
Sum	1.20	1.14	1.35	1.23	0.09
*B. pumilus* SAFR-032					
KatX2 1	0.11	0.12	0.12	0.12	0.00
KatX2 2	0.10	0.11	0.11	0.11	0.00
KatX2 3	0.09	0.09	0.09	0.09	0.00
KatX2 4	0.37	0.40	0.38	0.38	0.01
Sum	0.68	0.71	0.69	0.69	0.01
*B. subtilis* P*katA::katX2*					
KatX2 1	0.06	0.06	0.06	0.06	0.00
KatX2 2	0.07	0.06	0.07	0.07	0.01
KatX2 3	0.22	0.18	0.24	0.21	0.03
KatX2 4	0.80	0.77	0.90	0.82	0.06
Sum	1.14	1.06	1.27	1.16	0.09

The values are given in % volume representing the percentage of the spot volume of an individual spot of the entire spot volume of all spots detected on a gel

**Fig. 3:**
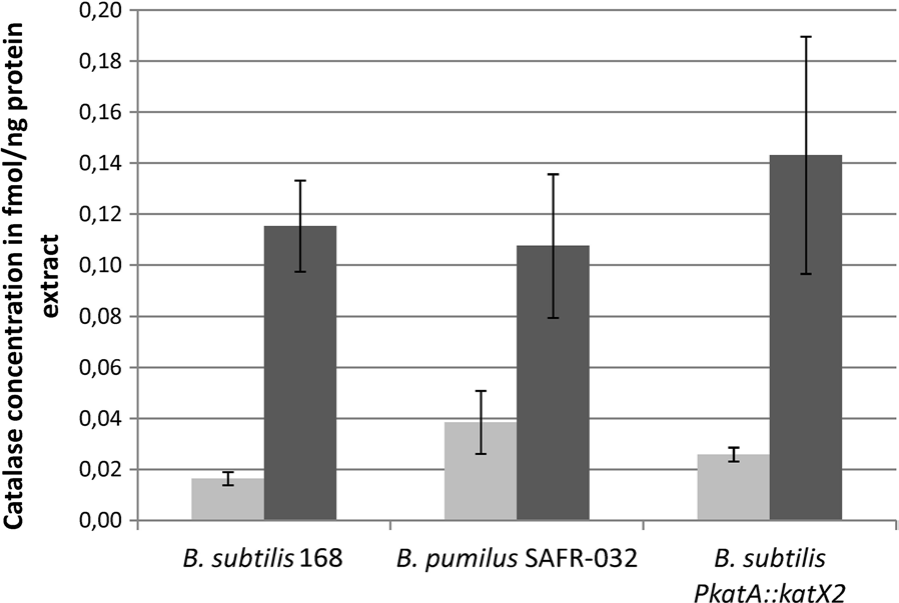
Concentration of catalase KatA in *B. subtilis* and KatX2 in *B. pumilus* and *B. subtilis* P*katA::katX2* under control conditions (*light grey*) and 20 min after addition of H_2_O_2_ (*dark grey*)

To gain information on the absolute concentration of the catalases in the cytoplasm, the gel- and label-free quantification approach LC-IMS^E^ was conducted. The results of this experiment revealed a concentration of KatA of 0.0165 fmol/ng protein extract in exponentially growing *B. subtilis* cells (Fig. 3). KatA accumulation increased up to 0.1153 fmol per ng protein extract in hydrogen peroxide stressed cells. In *B. pumilus* we measured an amount of 0.0385 fmol KatX2 per ng protein extract. 20 min after addition of H_2_O_2_, catalase accumulation increased up to 0.1076 fmol per ng cell extract in *B. pumilus* cells. Based on the lower basal accumulation the induction rate following H_2_O_2_ treatment was significantly higher in *B. subtilis* cells.

Exponentially growing *B. subtilis* P*katA::katX2* mutant cells contained 0.0259 fmol KatX2 catalase per ng protein extract (Fig. 3). Following hydrogen peroxide treatment the KatX2 accumulation increased up to 0.1431 fmol per ng cell extract and therefore it was higher than the KatX2 accumulation in H_2_O_2_ stressed *B. pumilus* cells.

### Catalase activity

The sequence differences between the KatX2 and the KatA-group may lead to a higher enzymatic activity and/or to a higher stability of the KatX2 enzyme, which could explain the enhanced hydrogen peroxide tolerance of *B. pumilus* compared to related organisms.

**Fig. 4:**
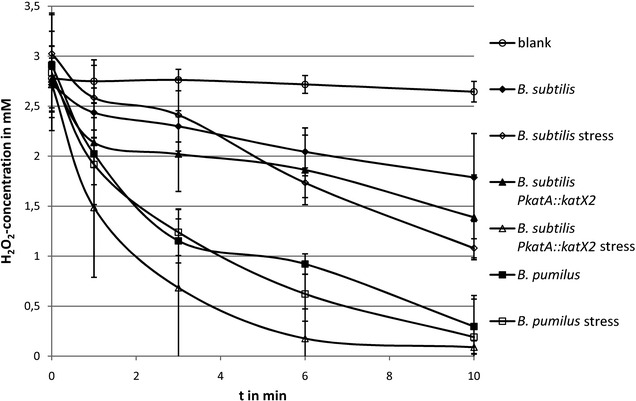
Degradation of H_2_O_2_ by cell extracts of *B. subtilis* 168, *B. subtilis* P*katA::katX2* mutant and *B. pumilus* SAFR-032. Extracts from exponentially growing cells are shown with *filled symbols*, extracts from cells previously stressed are shown with *empty symbols*

We used cell extracts containing equal amounts of protein to analyze the degradation of H_2_O_2_ by the catalases. For this we prepared extracts from exponentially growing cells as well as from cells previously stressed by low amounts of H_2_O_2_ (5 µM for *B. subtilis*, 200 µM for *B. pumilus*) (Fig. 4) [8, 11, 12]. As expected, the added H_2_O_2_ concentration was reduced faster in pre-stressed extracts. In both cases, the *B. pumilus* cell extract degraded the hydrogen peroxide faster than the extracts from *B*. *subtilis*. *B. subtilis* protein extracts degraded only about 50% of the added H_2_O_2_ within 10 min, whereas 80–90% of it was degraded by the *B. pumilus* extracts. The faster degradation of hydrogen peroxide by control cell extracts of *B. pumilus* could be explained by the higher catalase amount present in theses extracts (Fig. 3). However, in stressed cells the amount of the catalase is comparable between *B. subtilis* and *B. pumilus*. Therefore, the faster degradation of hydrogen peroxide by extracts from stressed *B. pumilus* cells cannot be due to a higher amount of the catalase. Using the absolute protein quantities determined with the LC-IMS^E^ method, we caculated specific enzyme activities for the catalases. For KatA a specific activity of 6.212 × 10^7^ units (µg/min) per mg catalase was determined. Specific activity of KatX2 from *B. pumilus* was 8.121 × 10^7^ units per mg catalase.

The hydrogen peroxide degradation of extracts from exponentially growing unstressed *B.* *subtilis* P*katA::katX2* mutant cells was comparable to those shown by the wild type. The specific activity was 8.628 × 10^7^ units per mg catalase. Pre-stressed extracts of the mutant cells degraded the added H_2_O_2_ much faster than the corresponding extracts from the other strains and organisms, even faster than the pre-stressed *B. pumilus* cell extracts (Fig. 4). This may be explained by the higher induction rate of the recombinant catalase KatX2 in *B. subtilis* following hydrogen peroxide treatment compared to the induction rate observed in stressed *B. pumilus*, which resulted in a slightly higher amount of the catalase in the cells (Fig. 3; Table 2) [8, 11].

### Modification of the KatX2 protein after peroxide stress

**Fig. 5:**
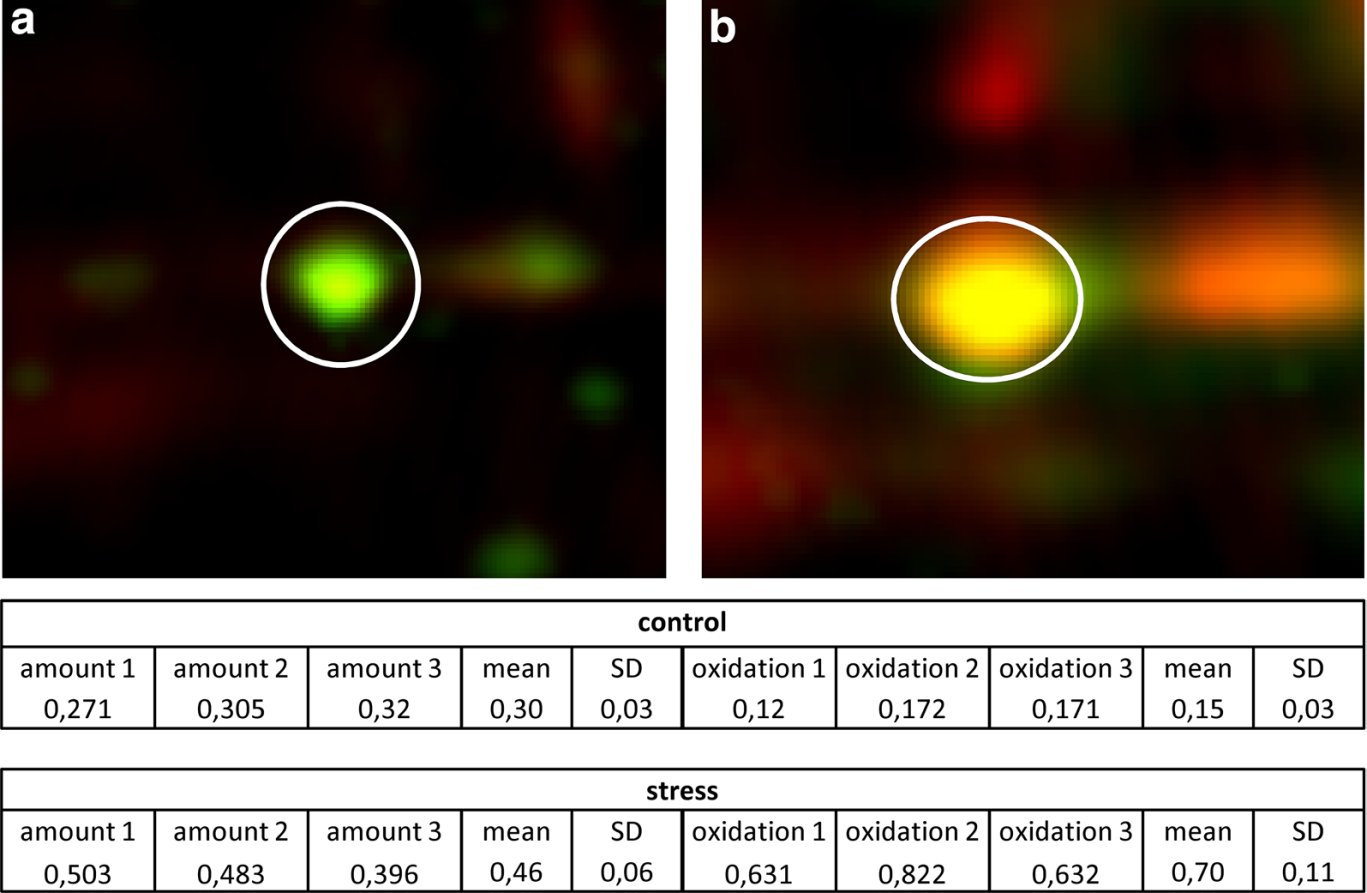
KatX2 protein spots in exponentially growing *B. pumilus* cells (**a**) and 20 min after the addition of 2 mM H_2_O_2_ (**b**). Protein accumulation is shown in *green*, reversible oxidized thiol-modifications stained with BODIPY fluorescent stain is shown in *red*. The table presents the relative spot volumes for three replicates. Amount: % vol of Flamingo stained accumulated protein spots, Oxidation: % vol of BODIPY stained oxidized protein spots

Unlike the KatA protein, KatX2 contains three cysteines. Under oxidative stress conditions cysteine residues can be oxidized [26, 27]. This could irreversibly damage the protein, e.g. when oxidized to cysteine sulfinic and sulfonic acid as shown for the GapA protein of *Staphylococcus aureus* [28]. To prevent this, *Bacillus* cells protect cysteine residues in proteins by reversibly oxidizing them using different low-molecular-weight thiol compounds such as bacillithiol [5, 29]. Using the fluorescence thiol modification assay described by Hochgräfe et al. [26] we analyzed reversible thiol-modifications in the *B. pumilus* KatX2 protein. This procedure uses two different staining methods, one for protein accumulation and one for reversible thiol oxidations. Quantification of proteins was done using relative spot volumes (volume of a spot compared to the volumes of all spots visible on the 2D-gel). The ratio between the spot volumes of a protein spot in the thiol modification staining and the protein accumulation staining is an indicator for the amount of reversible oxidations of the cysteine residues in a protein. In exponentially growing cells, KatX2 cysteine residues were nearly completely reduced (Fig. 5a). Hydrogen peroxide treatment caused a significant increase of reversible cysteine oxidation (Fig. 5b). The ratio of thiol modification to protein accumulation increased from about 0.5 to 1.36.

**Fig. 6:**
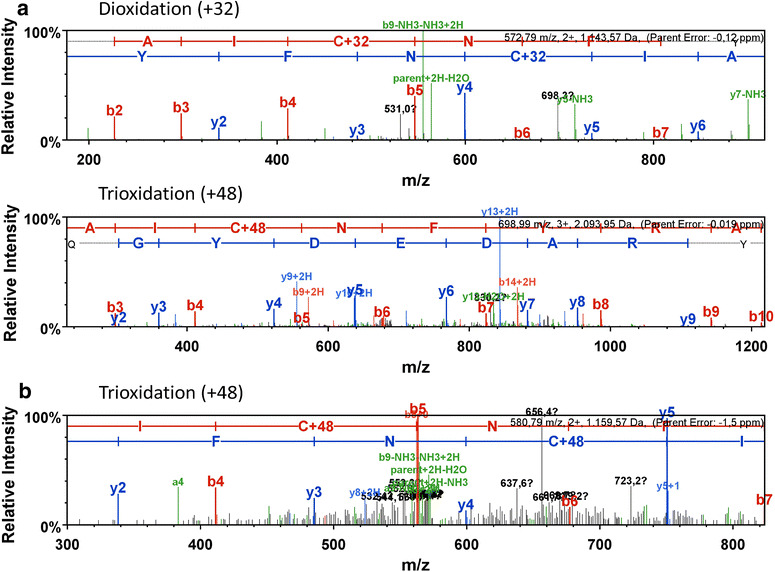
Spectra of modified cystein-containing peptides of KatX2 detected in the *B. subtilis* P*katA::katX2* mutant (**a**) and the *B. pumilus* strain (**b**)

When the oxidative stress is too harsh or continues too long, the capacity of the cells to protect proteins by reversible thiol oxidations might get exhausted leading to irreversible oxidation of cysteine residues to cysteine sulfinic and/or sulfonic acid. To test this hypothesis, LC–MS/MS analysis was performed to search for such irreversible oxidation of cysteine residues in the different catalase spots excised from the 2D-gels. Modifications were verified by analysing fragment ions of cysteine containing peptides. We found evidence for the oxidation of the thiol group of cysteine 461 in one of the catalase 2D-gel spots. Mass shifts of +32 and +48 were detected in KatX2 expressed by the *B. subtilis* P*katA::katX2* mutant representing sulfinic (+32) and sulfonic (+48) acid formation (Fig. 6a). In *B. pumilus* we only detected formation of sulfonic acid in the KatX2 cysteine 461 (Fig. 6b).

## Concluding remarks

By exchanging the *B. subtilis* KatA catalase with *B. pumilus* KatX2, we could show that this catalase enables a higher resistance of *B. subtilis* to oxidative stress. The *B. subtilis* P*katA::katX2* mutant showed an increased growth compared to the *B. subtilis* wild type when treated with hydrogen peroxide. However, H_2_O_2_ concentrations comparable to those used in *B. pumilus* studies nearly completely ceased growth of the *B. subtilis* P*katA::katX2* mutant. This fact points to further features of *B. pumilus* like a better protection of its proteins or a more effective machinery for repair of damaged compounds which are responsible for the enhanced resistance to hydrogen peroxide beside the high catalase activity of KatX2. Previous studies have demonstrated that oxidative stress indeed can lead to oxidation of secreted proteins which are often produced with microbial hosts [12]. *B. pumilus katX2* catalase might be an interesting marker gene for the engineering of new production strains which confer an enhanced resistance against hydrogen peroxide. Improving resistance of production strains to upcoming oxidative stress during fermentations has already been tried by mutating a catalase to increase catalytic efficiency [30]. This could ensure an improved quality of overproduced target proteins in bacterial production hosts.

### Additional file

**Additional file 1.** Schematic depiction of the steps leading to the linear DNA strand used for transformation in *B. subtilis* and the resulting situation in *B. subtilis* P*katA::katX2*.

## References

[1] Schweder T, Hecker M (2004). Monitoring of stress responses. Adv Biochem Eng Biotechnol.

[2] Stadtman ER, Levine RL (2003). Free radical-mediated oxidation of free amino acids and amino acid residues in proteins. Amino Acids.

[3] Farr SB, Kogoma T (1991). Oxidative stress responses in *Escherichia coli* and *Salmonella typhimurium*. Microbiol Rev.

[4] Imlay JA, Fridovich I (1991). Assay of metabolic superoxide production in *Escherichia coli*. J Biol Chem.

[5] Newton GL, Rawat M, La Clair JJ, Jothivasan VK, Budiarto T (2009). Bacillithiol is an antioxidant thiol produced in Bacilli. Nat Chem Biol.

[6] Enfors SO, Jahic M, Rozkov A, Xu B, Hecker M (2001). Physiological responses to mixing in large scale bioreactors. J Biotechnol.

[7] Schweder T, Krüger E, Xu B, Jürgen B, Blomsten G (1999). Monitoring of genes that respond to process-related stress in large-scale bioprocesses. Biotechnol Bioeng.

[8] Handtke S, Schroeter R, Jürgen B, Methling K, Schlüter R (2014). *Bacillus pumilus* reveals a remarkably high resistance to hydrogen peroxide provoked oxidative stress. PLoS ONE.

[9] Kempf MJ, Chen F, Kern R, Venkateswaran K (2005). Recurrent isolation of hydrogen peroxide-resistant spores of *Bacillus pumilus* from a spacecraft assembly facility. Astrobiology.

[10] Garcia-Ramon DC, Molina CA, Osuna A, Vilchez S (2016). An in-depth characterization of the entomopathogenic strain *Bacillus pumilus* 15.1 reveals that it produces inclusion bodies similar to the parasporal crystals of *Bacillus thuringiensis*. Appl Microbiol Biotechnol.

[11] Mostertz J, Scharf C, Hecker M, Homuth G (2004). Transcriptome and proteome analysis of *Bacillus subtilis* gene expression in response to superoxide and peroxide stress. Microbiology.

[12] Schroeter R, Voigt B, Jürgen B, Methling K, Pöther DC (2011). The peroxide stress response of *Bacillus licheniformis*. Proteomics.

[13] Gioia J, Yerrapragada S, Qin X, Jiang H, Igboeli OC (2007). Paradoxical DNA repair and peroxide resistance gene conservation in *Bacillus pumilus* SAFR-032. PLoS ONE.

[14] Kunst F, Ogasawara N, Moszer I, Albertini AM, Alloni G (1997). The complete genome sequence of the gram-positive bacterium *Bacillus subtilis*. Nature.

[15] Burkholder PR, Giles NH (1947). Induced biochemical mutations in *Bacillus subtilis*. Am J Bot.

[16] Benson AK, Haldenwang WG (1993). Regulation of sigma B levels and activity in *Bacillus subtilis*. J Bacteriol.

[17] Wach A (1996). PCR-synthesis of marker cassettes with long flanking homology regions for gene disruptions in *S. cerevisiae*. Yeast.

[18] Reder A, Gerth U, Hecker M (2012). Integration of sigmaB activity into the decision-making process of sporulation initiation in *Bacillus subtilis*. J Bacteriol.

[19] Reder A, Hoper D, Weinberg C, Gerth U, Fraunholz M (2008). The Spx paralogue MgsR (YqgZ) controls a subregulon within the general stress response of *Bacillus subtilis*. Mol Microbiol.

[20] Büttner K, Bernhardt J, Scharf C, Schmid R, Mäder U (2001). A comprehensive two-dimensional map of cytosolic proteins of *Bacillus subtilis*. Electrophoresis.

[21] Görg A, Boguth G, Obermaier C, Posch A, Weiss W (1995). Two-dimensional polyacrylamide gel electrophoresis with immobilized pH gradients in the first dimension (IPG-Dalt): the state of the art and the controversy of vertical versus horizontal systems. Electrophoresis.

[22] Wolf C, Hochgräfe F, Kusch H, Albrecht D, Hecker M (2008). Proteomic analysis of antioxidant strategies of *Staphylococcus aureus*: diverse responses to different oxidants. Proteomics.

[23] Muntel J, Fromion V, Goelzer A, Maabeta S, Mäder U (2014). Comprehensive absolute quantification of the cytosolic proteome of *Bacillus subtilis* by data independent, parallel fragmentation in liquid chromatography/mass spectrometry (LC/MS(E)). Mol Cell Proteom.

[24] Rappsilber J, Mann M, Ishihama Y (2007). Protocol for micro-purification, enrichment, pre-fractionation and storage of peptides for proteomics using StageTips. Nat Protoc.

[25] Ma Q, Wood TK (2011). Protein acetylation in prokaryotes increases stress resistance. Biochem Biophys Res Commun.

[26] Hochgräfe F, Mostertz J, Albrecht D, Hecker M (2005). Fluorescence thiol modification assay: oxidatively modified proteins in *Bacillus subtilis*. Mol Microbiol.

[27] Di Simplicio P, Franconi F, Frosali S, Di Giuseppe D (2003). Thiolation and nitrosation of cysteines in biological fluids and cells. Amino Acids.

[28] Weber H, Engelmann S, Becher D, Hecker M (2004). Oxidative stress triggers thiol oxidation in the glyceraldehyde-3-phosphate dehydrogenase of *Staphylococcus aureus*. Mol Microbiol.

[29] Lee JW, Soonsanga S, Helmann JD (2007). A complex thiolate switch regulates the *Bacillus subtilis* organic peroxide sensor OhrR. Proc Natl Acad Sci USA.

[30] Cao WK, Kang Z, Liu S, Liu L, Du G, Chen J (2014). Improved catalytic efficiency of catalase from *Bacillus subtilis* byrational mutation of Lys114. Process Biochem.

