# Supplementary figures and images for: Bacillus pumilus KatX2 confers enhanced hydrogen peroxide resistance to a Bacillus subtilis PkatA::katX2 mutant strain
Source: Microb Cell Fact. 2017 Apr 26;16:72. doi: 10.1186/s12934-017-0684-y (PMC5406934; doi:10.1186/s12934-017-0684-y)

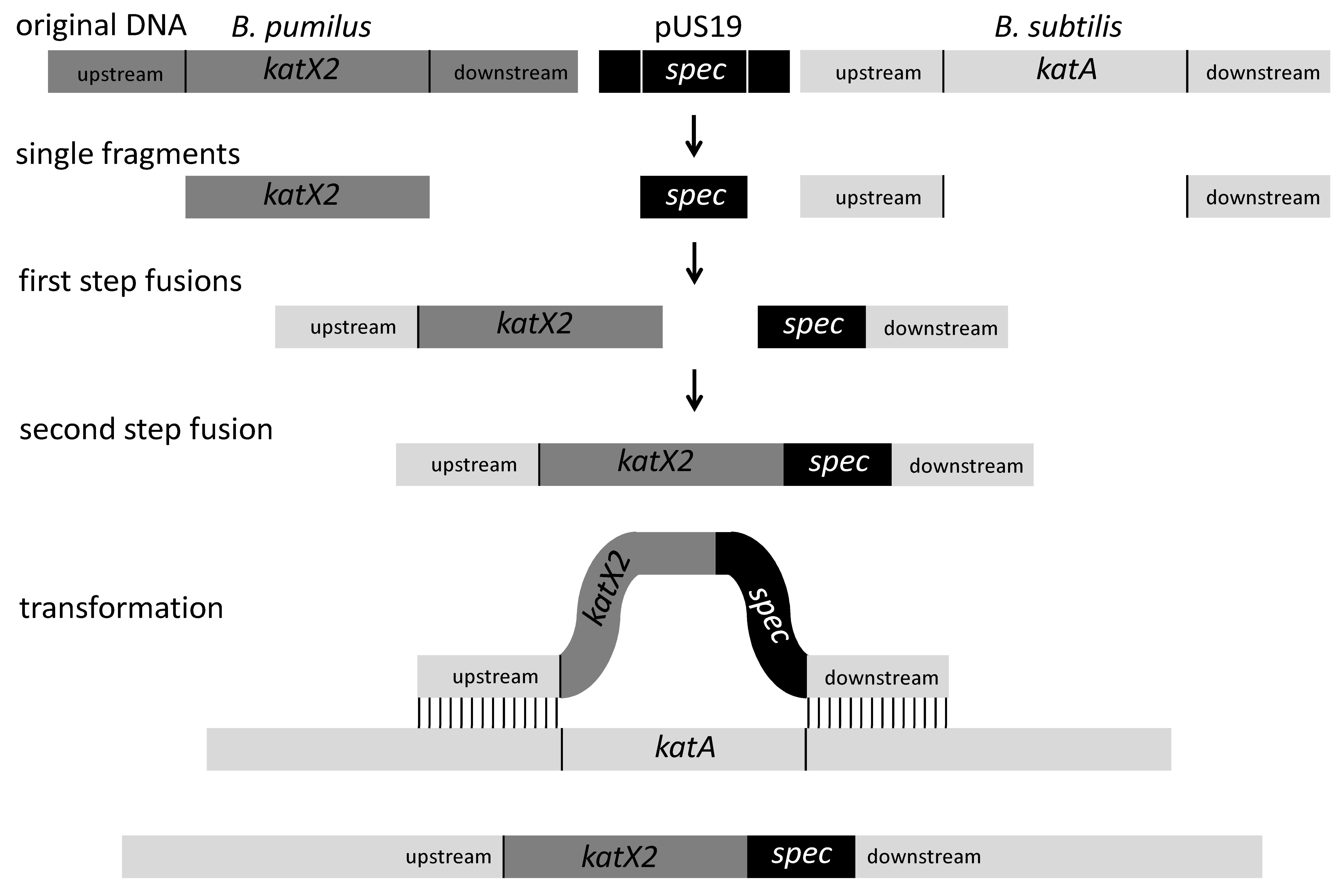

Supplement: Supplementary file 1 — Additional file 1. Schematic depiction of the steps leading to the linear DNA strand used for transformation in B. subtilis and the resulting situation in B. subtilis PkatA::katX2. [file 12934_2017_684_MOESM1_ESM.jpg]
